# Antitumor effect of anti-vascular therapy with STING agonist depends on the tumor microenvironment context

**DOI:** 10.3389/fonc.2023.1249524

**Published:** 2023-08-15

**Authors:** Justyna Czapla, Alina Drzyzga, Sybilla Matuszczak, Tomasz Cichoń, Marek Rusin, Magdalena Jarosz-Biej, Ewelina Pilny, Ryszard Smolarczyk

**Affiliations:** Center for Translational Research and Molecular Biology of Cancer, Maria Sklodowska-Curie National Research Institute of Oncology, Gliwice, Poland

**Keywords:** STING signaling, anti-vascular therapy, immunotherapy, immune checkpoint inhibitors, the tumor microenvironment

## Abstract

**Introduction:**

Targeting tumor vasculature is an efficient weapon to fight against cancer; however, activation of alternative pathways to rebuild the disrupted vasculature leads to rapid tumor regrowth. Immunotherapy that exploits host immune cells to elicit and sustain potent antitumor response has emerged as one of the most promising tools for cancer treatment, yet many treatments fail due to developed resistance mechanisms. Therefore, our aim was to examine whether combination of immunotherapy and anti-vascular treatment will succeed in poorly immunogenic, difficult-to-treat melanoma and triple-negative breast tumor models.

**Methods:**

Our study was performed on B16-F10 melanoma and 4T1 breast tumor murine models. Mice were treated with the stimulator of interferon genes (STING) pathway agonist (cGAMP) and vascular disrupting agent combretastatin A4 phosphate (CA4P). Tumor growth was monitored. The tumor microenvironment (TME) was comprehensively investigated using multiplex immunofluorescence and flow cytometry. We also examined if such designed therapy sensitizes investigated tumor models to an immune checkpoint inhibitor (anti-PD-1).

**Results:**

The use of STING agonist cGAMP as monotherapy was insufficient to effectively inhibit tumor growth due to low levels of STING protein in 4T1 tumors. However, when additionally combined with an anti-vascular agent, a significant therapeutic effect was obtained. In this model, the obtained effect was related to the TME polarization and the stimulation of the innate immune response, especially activation of NK cells. Combination therapy was unable to activate CD8^+^ T cells. Due to the lack of PD-1 upregulation, no improved therapeutic effect was observed when additionally combined with the anti-PD-1 inhibitor. In B16-F10 tumors, highly abundant in STING protein, cGAMP as monotherapy was sufficient to induce potent antitumor response. In this model, the therapeutic effect was due to the infiltration of the TME with activated NK cells. cGAMP also caused the infiltration of CD8^+^PD-1^+^ T cells into the TME; hence, additional benefits of using the PD-1 inhibitor were observed.

**Conclusion:**

The study provides preclinical evidence for a great influence of the TME on the outcome of applied therapy, including immune cell contribution and ICI responsiveness. We pointed the need of careful TME screening prior to antitumor treatments to achieve satisfactory results.

## Introduction

Therapies that target tumor vasculature are efficient in tumor burden reduction. Vascular disrupting agents (VDAs) induce apoptosis of tumor endothelial cells for example by affecting the microtubule polymerization stability. Destruction of endothelial cells leads to vascular system disruption and finally causes tumor cell necrosis in the tumor tissue (1, 2). VDAs also exert indirect effects by immune stimulation. It has been shown that VDAs have potential to convert “immunologically cold” into “immunologically hot” tumors (3). However, after initial tumor reduction, its rapid regrowth is observed due to viable rim cells remaining at the periphery of the tumor (1). Therefore, combining VDAs with additional immunostimulation seems a rationale to awaken potent antitumor immune response to eradicate remaining cancer cells. However, it must also be taken into account that only the proper administration sequence of the immunostimulants and other anticancer agents causes a synergistic antitumor effect. For example, a combination in which tumors were irradiated prior to VDA administration effectively inhibited melanoma growth, whereas a reverse combination of therapeutic agents showed no therapeutic effect and even abolished the effect (4).

Immunotherapy has emerged as one of the most promising cancer treatments. Immunotherapy covers five main fields: checkpoint blockade therapy, adoptive cell immunotherapy, oncolytic immunotherapy, cancer vaccines, and immunostimulating/cytokine therapy (5). Despite tremendous progress in immunotherapy over the recent years, most patients do not respond or develop resistance mechanisms to applied treatment. Most immunotherapies are aimed at CD8^+^ T-cell response augmentation. However, the poor CD8^+^ T-cell response is mainly due to lack of neoantigens for T-cell recognition, incomplete antigen presentation, and loss of major histocompatibility complex (MHC) class I. A sustained immunosuppressive tumor microenvironment (TME) also contributes to immunotherapy resistance. Thus, therapies mobilizing various effector cells other than CD8^+^ T cells are needed to effectively cure difficult-to-treat, refractory tumors.

The cyclic GMP–AMP synthase (cGAS)–stimulator of interferon gene (STING) pathway senses cytosolic DNA resulting in the production of multiple inflammatory mediators, including type I interferons and proinflammatory cytokines. It activates the innate immune system to fight against viruses or bacterial infections. The cGAS–STING pathway is naturally activated in tumors, but its antitumor response is rather weak. The cyclic GMP-AMP (cGAMP) dinucleotide is a natural STING protein activator synthesized by cGAS synthase following cytosolic DNA binding. Natural or synthetic cyclic dinucleotides (CDNs), injected systemically or intratumorally, serve as powerful STING agonists. CDNs mobilize potent antitumor response by both CD8^+^ T cells and NK cells. Activation of the cGAS-STING pathway as immunotherapy has shown promising results, including long-term remissions in some preclinical tumor models (6, 7). However, in many refractory tumor models, targeting the STING protein results in insufficient or unsustainable antitumor response. It may be due to the low expression of the STING protein in tumors, lack of T-cell epitopes or MHC I molecules, or immunosuppressive tumor milieu. Therefore, adequate combination therapy is required to activate stronger antitumor response.

In this study, we sought to investigate whether a combination of a vascular disruption agent (combretastatin A4 phosphate—CA4P) and a cGAS-STING pathway activator (cGAMP) could lead to an increased therapeutic potential in targeting two poorly immunogenic, difficult-to-treat MHC I-deficient (B16-F10 melanoma) and MHC I-positive (4T1 breast cancer) tumor models and, if it is so, which immune cells are mainly involved in cancer cell eradication. We also explored if such a combination could reverse the resistance to anti-PD-1 therapy. This study provides preclinical evidence of the importance of the tumor microenvironment status when designing novel combination therapies.

## Materials and methods

Details of material and methods are described in the **Supplementary Materials**.

### Cell lines

The murine melanoma B16-F10 cell line and 4T1 breast cancer cells (ATCC, Manassas, WV, USA) were cultured in RPMI 1640 (Thermo Fisher Scientific, Waltham, MA, USA) supplemented with 10% heat-inactivated fetal bovine serum (Thermo Fisher Scientific).

### Western blot analysis

The cells were lysed with the IP buffer supplemented with protease and phosphatase inhibitors. Lysates were separated by SDS-PAGE and electro-transferred onto PVDF membranes. The membranes were blocked and then incubated with the following primary antibodies: anti-STING (clone: D2P2F, Cell Signaling Technology, Danvers, MA, USA) and anti-HSC70 (clone: B-6, Santa Cruz Biotechnology, Dallas, TX, USA). HRP-conjugated secondary antibodies were detected by chemiluminescence (Thermo Fisher Scientific).

### Therapeutic agents

Cyclic guanosine monophosphate–adenosine monophosphate (cGAMP, InvivoGen, Toulouse, France) was injected intratumorally in a dose of 5 µg/mouse (in 100 µl of PBS¯). Combretastatin A4 phosphate disodium (CA4P, Selleckchem, Planegg, Germany) was injected intraperitoneally at a dose of 50 mg/kg body weight in 100 µl saline/mouse. A monoclonal anti-PD-1 antibody (clone: RMP1-14, BioLegend, San Diego, CA, USA) in a dose of 200 µg in 100 µl PBS¯/mouse was injected intraperitoneally.

### Therapy

**Figure 1:**
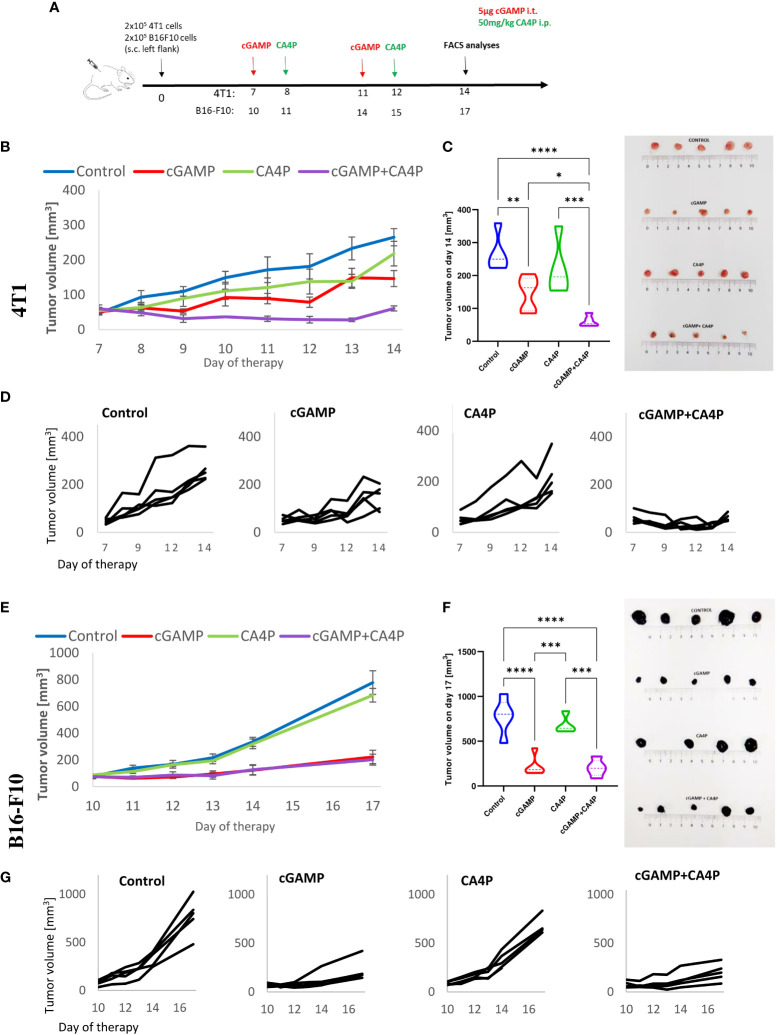
Inhibition of murine breast tumor (4T1) and melanoma (B16-F10) growth using combination therapy of STING agonist and anti-vascular agent. **(A)**Diagram depicting the treatment schedule of a STING agonist (cGAMP) in combination with an anti-vascular agent (CA4P) in subcutaneous (s.c.) breast tumor of BALB/c mice and melanoma of C57Bl/6NCrl mice. Black arrows indicate cGAMP treatment, red arrows CA4P treatment. **(B, E)** Tumor volume was measured with a caliper every 1 or 2 days (mean ± SEM). **(C, F)** Diagram and pictures showing tumor volume on the last day of experiment. **(D, G)** Plots showing individual tumor growth in each treatment group. Data are representative of three independent experiments, n = 5 in each group. Statistical analysis was performed on the last day of experiment, **(C)** ANOVA with post-hoc LSD test. **(F)** Tukey HSD test for unequal N. *p < 0.05, **p < 0.01, ***p < 0.001, ****p < 0.0001.

**Figure 2:**
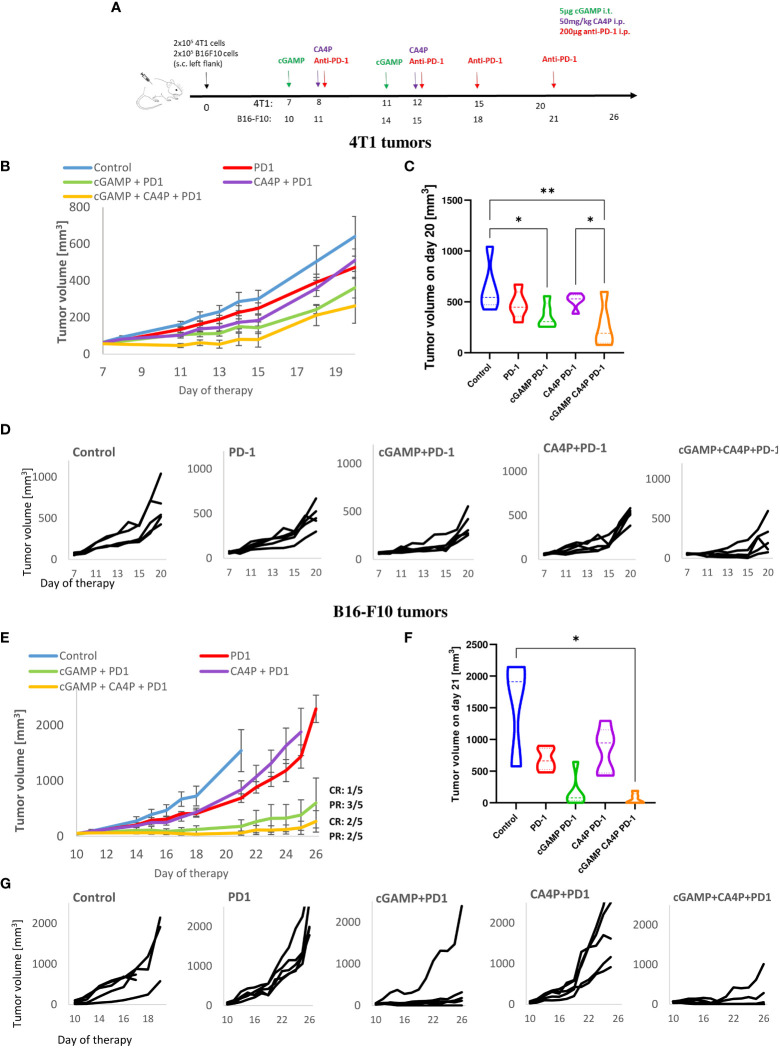
Inhibition of murine breast tumor (4T1) and melanoma (B16-F10) growth using combination therapy of STING agonist, anti-vascular agent and immune checkpoint inhibitor. **(A)** Diagram depicting the treatment schedule of STING agonist (cGAMP) in combination with anti-vascular agent (CA4P) and immune checkpoint inhibitor (anti-PD-1 antibody) in subcutaneous (s.c.). breast tumor of BALB/c mice and melanoma of C57Bl/6NCrl mice. Black arrows indicate cGAMP treatment, red arrows CA4P treatment, green arrows anti-PD-1 treatment. **(B, D)** Tumors volume was measured with calliper every one or two days (mean ± SEM). **(C, F)** Diagram showing tumors volume on the last day of experiment. **(D, G)** Plots showing individual tumor growth in each treatment group. Data are representative of pilot experiment, n=5 in each group. Statistical analysis was performed on the last day of experiment. Data are shown as **(C)** mean ± SEM for *p<0.05, **p<0.01, by Anova with post-hock LSD Test or **(E)** median ± interquartile range for *p<0.05, by Kruskal–Wallis multiple comparisons.

C57Bl/6NCrl or BALB/c mice were injected subcutaneously into the lower flank with 2 × 10^5^ B16-F10 cells or 2 × 10^5^ 4T1 cells in 100 µl PBS¯, respectively. Tumors were measured with calipers, and tumor volumes were determined using the formula volume = width^2^ × length × 0.52. When tumors reached approximately 50 mm^2^ (7 days after inoculation), the animals were treated according to group allocation: the control group left untreated, the cGAMP group with two doses of cGAMP in a 4-day interval, the CA4P group two doses of CA4P in a 4-day interval, and the cGAMP + CA4P group with two doses of cGAMP and CA4P in a 4-day interval with a 1-day shift. In an experiment, combination of immune checkpoint inhibitor and anti-PD-1 antibody was administered three times (in BALB/c mice) and four times (in C57Bl/6NCrl mice) in 3- to 4-day intervals. The schedule of therapeutic agent administration is shown in **Figures 1**, **2**.

### Immunofluorescence staining

The collected tumors were embedded in OCT (Leica Biosystems, Wetzlar, Germany), frozen in liquid nitrogen, and sectioned into 5-µm slices. Blood vessels were stained with anti-CD31 antibody (Abcam; Cambridge, UK). Macrophages in the tumor sections were stained with anti-F4/80 (Abcam) and anti-CD206 antibodies (BioLegend). Activated NK cells were identified using anti-NKp46 antibody (BioLegend). The obtained confocal images were analyzed with ImageJ 1.48v (NIH, Bethesda, MD, USA), and the results were expressed as the percentage of area (%).

### Flow cytometry analysis

The subpopulations of T lymphocytes were identified using anti-CD45, anti-CD8, anti-CD69, and anti-PD-1 antibodies (BioLegend). The level of NK cells was determined using anti-CD45, anti-CD49b, anti-NKp46, anti-CD69 antibodies (BioLegend). Macrophages were identified using anti-CD11b, anti-F4/80, anti-CD86, and anti-CD206 antibodies (BioLegend). To analyze macrophage polarization, viable tumor-derived CD11b^+^ F4/80^+^ cells (to identify TAMs) were gated and then CD86 with CD206 antigens were assessed (to identify M1 macrophage phenotype: CD206^-^CD86^+^ cells, M2 macrophage phenotype: CD206^+^CD86^-^, and “transition M1↔M2” macrophages (CD206^+^CD86^+^ cells). Dead cells were eliminated by using the viability dye 7-AAD (BioLegend). In flow cytometric analyses (BD FACSCanto; BD, Franklin Lakes, NJ, USA), gates dividing negative from positive cells were based on isotype antibody control probes.

### Statistics

For each group of variables, the normality of distribution and homogeneity of variance were verified. Statistical analysis was conducted using analysis of variance (ANOVA) with *post-hoc* Tukey HSD or LSD tests or using the Kruskal–Wallis test. p value <0.05 was considered statistically significant.

## Results

### Differential expression of STING protein and MHC class I molecule in breast tumor and melanoma

**Figure 3:**
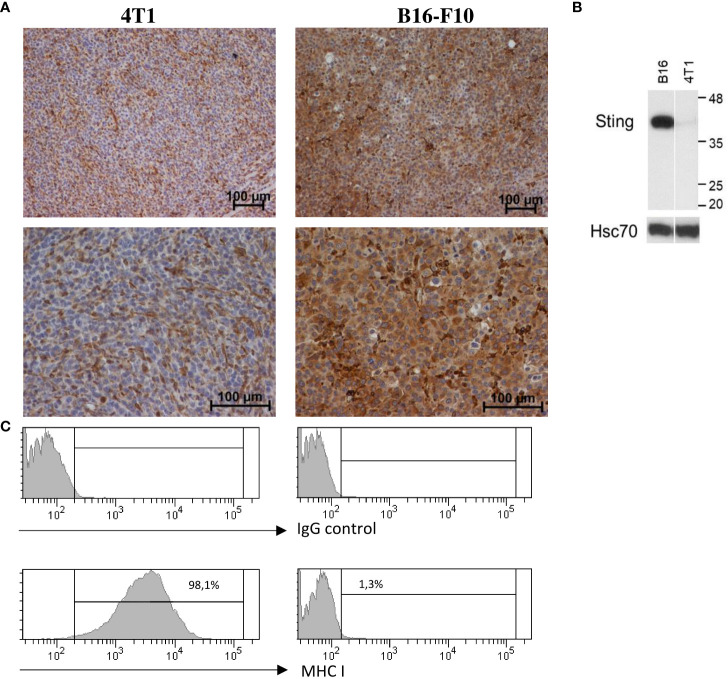
STING and MHC-I expression in murine breast carcinoma (4T1) and melanoma (B16-F10). **(A)** Immunohistochemical analysis was performed to visualize STING expression patterns in 4T1 and B16-F10 tumors. Magnification 100× and 200×, n = 3. **(B)** Total protein was extracted from 4T1 and B16-F10 cell lines, and the level of STING was detected by using western blotting. Hsc70 was used as a loading control, n = 3. **(C)** The expression of MHC class I on 4T1 and B16-F10 cell lines was determined by flow cytometry, n = 3. One representative experiment in each picture is shown.

In order to estimate the response rate to the STING agonist, we examined the expression level of the STING protein in murine 4T1 breast cancer and B16-F10 melanoma cell lines and in respective tumors. Western blot analysis showed an elevated level of STING protein in the B16-F10 cell protein extract, whereas in the 4T1 protein extract, only trace amounts were detected (**Figure 3B**). IHC analysis performed in well-developed tumors showed that in 4T1 tumors, STING protein was expressed by cells of tumor stroma (fibroblast-like cells) and endothelial cells. In B16-F10 tumors, STING protein was abundantly expressed by cancer cells (**Figure 3A**). We also assessed MHC class I expression in cancer cell lines. Flow cytometry analysis revealed that all 4T1 cells express MHC class I whereas B16-F10 do not express any (**Figure 3C**).

Therefore, our study was conducted further on two tumor models, which would provide mechanistically different antitumor immune responses.

### Combination immunotherapy of cGAMP and CA4P shows a synergistic antitumor effect against 4T1 breast cancer

Mice with 4T1 tumors were treated with cGAMP and CA4P (as shown in the diagram of **Figure 1A**). Neither intratumoral cGAMP nor intraperitoneal CA4P monotherapy led to spectacular tumor growth delay; however, cGAMP was slightly more effective (**Figure 1B**). In contrast to this, combination therapy resulted in a superior inhibitory effect on 4T1 tumor growth (**Figures 1B, C**). On day 14, the tumor volume in the combination group was about 2.5 times smaller than in the cGAMP group (~60 vs 150 mm^3^), 3.5 times smaller in the CA4P group (~60 vs 220 mm^3^), and about four times smaller than in the control group (~60 vs 260 mm^3^) (**Figure 1B**).

### CA4P does not provide an additive antitumor effect in combination with cGAMP against B16-F10 melanoma

Mice with B16-F10 melanoma tumors () were treated as shown in the diagram (**Figure 1A**). CA4P monotherapy did not show any therapeutic effect (**Figure 1E**). cGAMP both as monotherapy and in combination with an anti-vascular agent caused the same B16-F10 tumor growth inhibition (**Figure 1F**). On day 17, the tumor volumes in the cGAMP monotherapy group and combination group were about seven times smaller than in the control and CA4P groups (100 vs 700 mm^3^) (**Figure 1E**). No additional benefit of the anti-vascular compound was observed in this type of tumor.

### Combination therapy effectively reduce tumor blood vessel density

**Figure 4:**
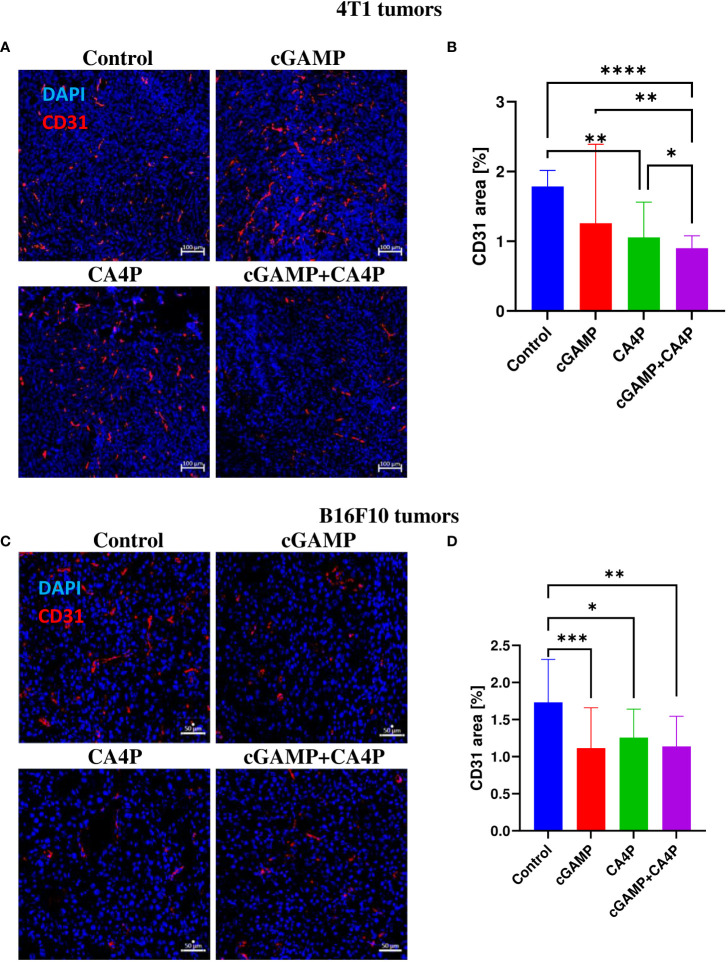
The effect of combination therapy on 4T1 **(A, B)** and B16-F10 **(C, D)** tumors vascularization. At the end of the therapy (as depicted on therapy scheme **Figure 3A**) mice were sacrificed and tumors were collected. **(A, C)** Tumor sections were stained with anti-CD31 antibody. CD31 positive endothelial cells (Alexa Fluor 594, red) and nuclei (DAPI, blue) were visualized using confocal microscope. Photographs were taken in 5-10 randomly chosen fields (magn. 200×) per section in 5 tumors of each group, one experiment. Representative photographs are shown. **(B, D)** Percentage of tumor area covered by blood vessels was calculated using ImageJ software. Data are shown as median ± interquartile range for *p < 0.05, **p < 0.01, ***p < 0.001, ****p < 0.0001 by Kruskal–Wallis multiple comparisons.

Considering the role of CA4P and cGAMP as agents affecting tumor blood vessels (8, 9), we examined the density of blood vessels. We observed that cGAMP induced vascular disruption at a similar level to CA4P in both tumor models. However, in 4T1 tumors, combination therapy led to significantly reduced tumor blood vessel density compared with the control and the CA4P and cGAMP groups. The area of blood vessels in 4T1 tumors was reduced by 30%, 40%, and 50% in the cGAMP, CA4P, and combination groups, respectively (**Figures 4A, B**). In B16-F10 tumors, the area covered by blood vessels was reduced following both cGAMP and CA4P administration, and also their combination at a similar level. The area of blood vessels in B16-F10 tumors was reduced in all treated groups of mice by 30%–37%. Statistical differences were observed between the control and each of the treated group (**Figures 4C, D**). The anti-vascular effect of cGAMP in combination with CA4P was only seen in 4T1 tumors but not in B16-F10 tumors.

### Combination immunotherapy of cGAMP and CA4P boost innate immunity in breast and melanoma tumors

**Figure 5:**
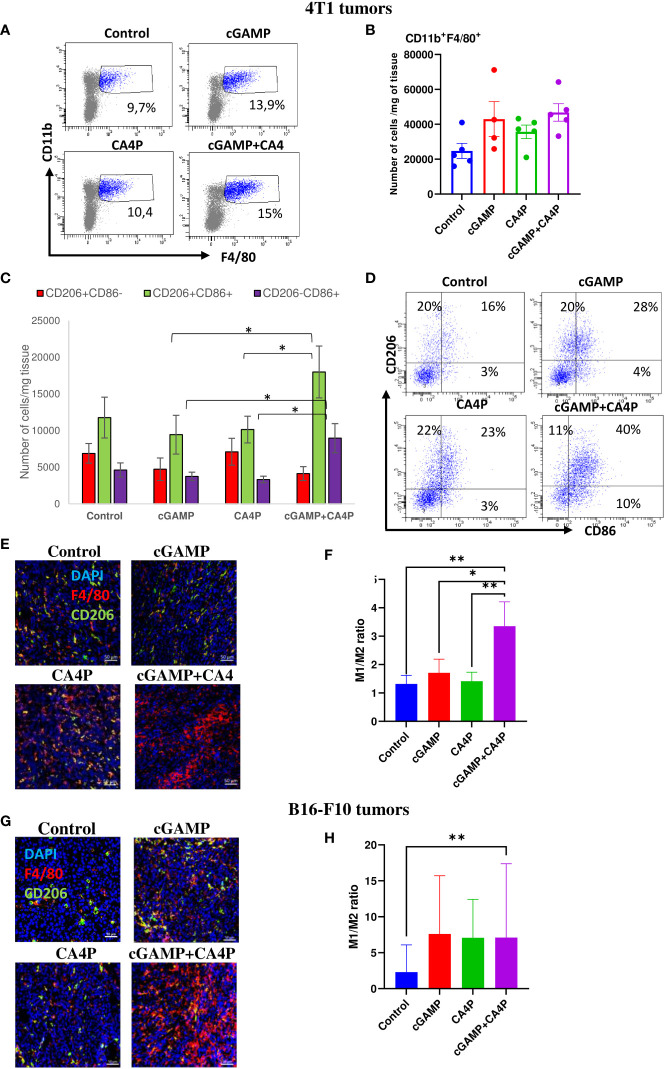
Tumor-associated macrophages (TAMs) infiltration within the tumor microenvironment in 4T1 **(A–F)** and B16-F10 **(G, H)** tumors following applied treatments. At the end of the therapy (as depicted on therapy scheme on **Figure 3A**) mice were sacrificed and tumors were collected for flow cytometry and IHC analysis. **(A–D)** Tumors were digested with 500U/mL of collagenase II and single cells suspension was stained with antibodies: CD11b, F4/80, CD206, CD86. TAMs were gated as alive 7-AAD^-^CD11b^+^F4/80^+^ cells. The percentage of CD206^+^ and/or CD86^+^ subpopulation of macrophages was determined from TAMs population gate. **(A)** TAMs were gated as alive CD11b^+^F4/80^+^ cells. **(B)** Total number of TAMs in the tumor **(C)** The number of CD11b^+^F4/80^+^ macrophages expressing CD86 (as M1 TAMs) or CD206 (as M2 TAMs) antigens from obtained total single cell-suspensions were calculated per 1 mg of tumor tissue. Data are shown as mean ± SEM for *p<0.05 by Anova with post-hock LSD Test or median ± interquartile range for *p<0.05, by Kruskal–Wallis multiple comparisons. **(D)** Representative flow data show polarization state of TAMs in respective groups. In all experimental groups selected cells were gated to appropriate isotype control for each sample individually. Results from three replicate experiments are shown, n=5 for each group. **(E, G)** Tumor sections were stained with anti-F4/80 antibody (Alexa Fluor 594, red), anti-CD206 (Alexa Fluor 488, green) and DAPI (blue). Sections were visualized using confocal microscope. Photographs were taken in 5-10 randomly chosen fields (magn. 200×) per section in 5 tumors of each group. Representative photographs are shown. **(F, H)** Percentage of tumor area covered by F4/80^+^ cells (as M1 TAMs) and CD206^+^ (as M2 TAMs) was calculated using ImageJ software. The ration of area covered by M1 to M2 TAMs is presented. Data are shown as mean ± SEM for *p<0.05, **p<0.01 by Anova with post-hock LSD Test **(F)** or median ± interquartile range for *p<0.05 by Kruskal–Wallis multiple comparisons **(H)**.

Since the STING pathway activates innate antitumor immunity, we assessed if combination therapy can favorably convert “cold,” poorly immunogenic TME into “hot” TME by shifting the tumor-associated macrophage (TAM) phenotype from protumorigenic M2 toward antitumorigenic M1. We assessed that well-developed breast tumor and melanoma established highly immunosuppressive milieu characterized by protumoral M2 macrophage infiltration (**Figures 5E, G**). Combination therapy led to a massive TAM infiltration in both tumor models (**Figures 5E, G**). We observed significant TME conversion in 4T1 tumors following combination of cGAMP and CA4P (**Figures 5A, B**). The number of M2 macrophages in the TME was slightly reduced in the cGAMP and the combination groups. On the other hand, the number of M1 macrophages and “transition M1↔M2” macrophages was significantly increased in the combination group compared with each of the study groups (**Figures 5C, D**). The ratio of M1 to M2 macrophages was over two times higher in the combination group compared with the other groups (**Figure 5F**). In B16-F10 tumors, the ratio of M1 to M2 macrophages was also significantly increased following combination therapy (more than a threefold increase when compared with the control group). Additionally, in each of the monotherapy groups, the ratio of M1/M2 was higher in comparison with the control group. However, only in the combination group was the M1/M2 ratio changed with statistical significance compared with the control group (**Figure 5H**).

**Figure 6:**
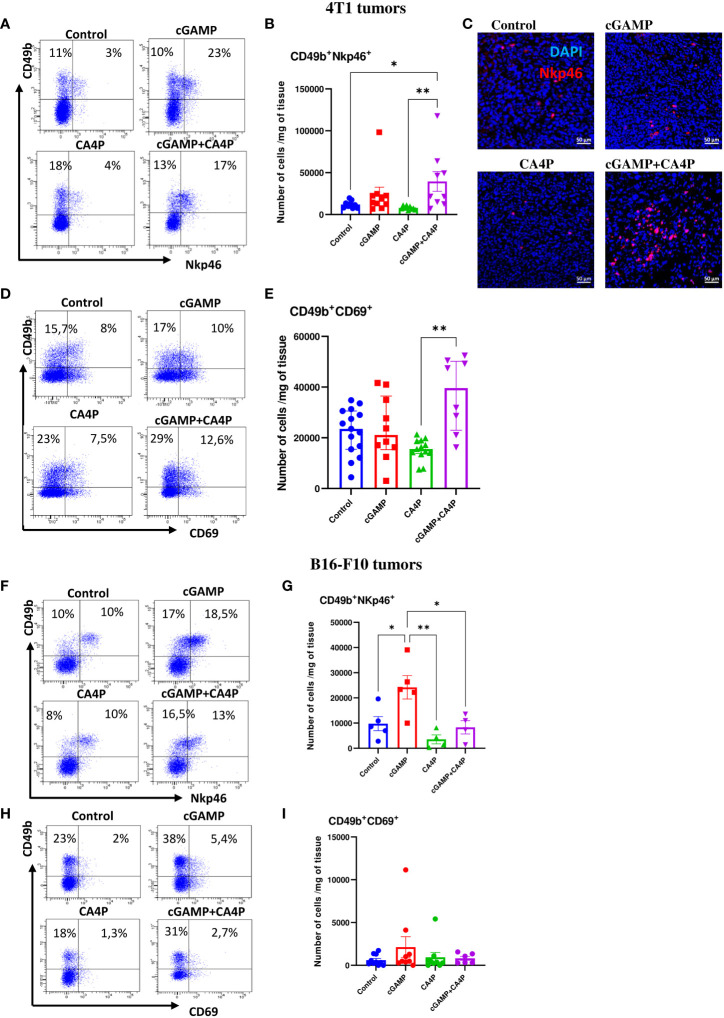
NK cells infiltration and activation within the tumor microenvironment in 4T1 **(A–E)** and B16-F10 **(F–I)** tumors following applied treatments. At the end of the therapy (as depicted on therapy scheme on **Figure 3A**) mice were sacrificed and tumors were collected for flow cytometry and IHC analysis. **(A, D, F, H)** Tumors were digested with 500U/mL of collagenase II and single cells suspension was stained with antibodies: CD45, CD49b, NKp46, CD69. Representative flow data show activation state of NK cells in respective groups. The percentage of CD49b^+^NKp46^+^ and CD49b^+^CD69^+^ subpopulation of NK cells was determined from the 7-AAD^-^CD45^+^ population gate. In all experimental groups selected cells were gated to appropriate isotype control for each sample individually. **(B, E, G, I)** The number of NK cells expressing activating receptors (CD49b^+^NKp46^+^ and CD49b^+^CD69^+^) from obtained total single cell-suspensions were calculated per 1 mg of tumor tissue. Results from three replicate experiments are shown, n=5-15 for each group. Data are shown as **(B, G)** mean ± SEM for *p<0.05, **p<0.01 by Anova with post-hock LSD Test or **(E)** median ± interquartile range for **p<0.01, by Kruskal–Wallis multiple comparisons. **(C)** Tumor sections were stained with anti-Nkp46 antibody (Alexa Fluor 594, red) and DAPI (blue). Sections were visualized using confocal microscope. Photographs were taken in 5-10 randomly chosen fields (magn. 200×) per section in 5 tumors of each group. Representative photographs are shown.

It has been shown that the immunosuppressive TME alters the NK-cell phenotype and activity causing NK cells to be dysfunctional. Thus, we assessed if reverting the TME following combination of cGAMP and CA4P enabled recruitment and activation of NK cells. We have shown that in the control 4T1 tumors, expressing MHC class I molecules, NK cells present within the TME were suppressed. They expressed low levels of NKp46- and CD69-activating receptors (**Figures 6A–E**) and were unable to reject tumors. Intratumoral cGAMP treatment induced NK-cell accumulation within tumors, but their significant activation occurred only when combination with CA4P was applied (**Figures 6B, E**). The number of activated NK cells was almost two times higher in the combination group compared with the cGAMP group (**Figures 6B, E**). In the control, MHC I-deficient B16-F10 tumors, the total number of activated NK cells within the TME was similar to 4T1 tumors which were also unable to reject tumors. Intratumoral cGAMP monotherapy was sufficient to induce massive NK-cell infiltration and a 2.5 times increase of NKp46- and a 1.5 times increase of CD69-activating receptors compared with the other groups. Unlike 4T1 tumors, combination with CA4P did not additionally enhance either NK-cell infiltration or their activation (**Figures 6F–I**).

These results indicate that combination of STING agonist and anti-vascular agent effectively converts the immunosuppressive TME in both tumor models through TAM polarization switch. Combination therapy activates antitumor NK-cell response in MHC-I^+^/STING^low^ (4T1) tumors, whereas in MHC I-deficient/STING^high^ (B16-F10) tumors, STING agonist as monotherapy provides sufficient anti-tumor NK cells response.

### Combination immunotherapy of cGAMP and CA4P rescue of exhausted CD8^+^ T cells

The existence of CD8^+^ cytotoxic T cells in tumor provides potent antitumor activity. However, immunosuppressive TME leads to CD8^+^ T-lymphocyte dysfunction and exhaustion through PD-1 expression. Unmasking CD8^+^ cytotoxic T-cell responses against tumor-derived antigens is crucial for effective immunotherapy.

**Figure 7:**
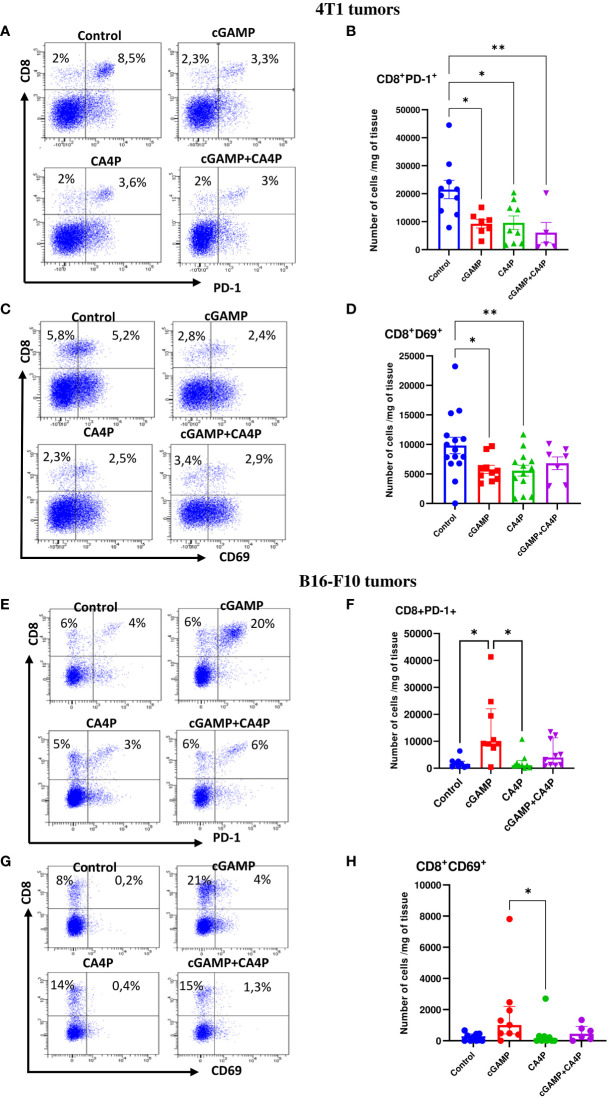
Characterization of exhausted CD8+ T cells phenotype within the tumor microenvironment in 4T1 **(A–D)** and B16-F10 **(E–H)** tumors following applied treatments. At the end of the therapy (as depicted on therapy scheme on **Figure 3A**) mice were sacrificed and tumors were collected for flow cytometry analysis. Tumors were digested with 500U/mL of collagenase II and single cells suspension was stained with antibodies: CD45, CD8, PD-1, CD69. **(A, C, E, G)** Representative flow data show expression of exhaustion markers on CD8^+^ T cells in respective groups. The percentage of CD8^+^PD-1^+^ and CD8^+^CD69^+^ subpopulation of CD8^+^ T cells was determined from the 7-AAD^-^CD45^+^ population gate. In all experimental groups selected cells were gated to appropriate isotype control for each sample individually. **(B, D, F, H)** The number of CD8^+^ T cells expressing markers of exhaustion (PD-1^+^, CD69^+^) from obtained total single cell-suspensions were calculated per 1 mg of tumor tissue. Results from three replicate experiments are shown, n=5-15 for each group. Data are shown as **(B, D)** mean ± SEM for *p<0.05, **p<0.01 by Anova with post-hock HSD Tuckey or LSD Tests or **(F, H)** median ± interquartile range for *p<0.05, by Kruskal–Wallis multiple comparisons.

In our study, we found that in well-developed 4T1 tumors, despite the large number of CD8^+^ T cells (**Supplementary Figure 1**), the majority of them expressed the PD-1 inhibitory receptor (**Figure 7A**). Intratumoral cGAMP treatment did not elevate the level of tumor-infiltrating CD8^+^ T cells as monotherapy or in CA4P combination (**Figure 7A**). However, applying each of the therapies resulted in a reduction of exhausted CD8^+^ T cells within tumor mass. The number of tumor-infiltrating CD8^+^PD-1^+^ T cells was approximately 3.5 times smaller following each of the applied treatment (**Figure 7B**). We found that expression of the CD69 marker, which was shown to play an important role in inducing the exhaustion of T cells (10), was also elevated in the control 4T1 tumors (**Figures 7C, D**). cGAMP and CA4P treatment significantly reduced the number of CD8^+^CD69^+^ T cells within the TME, while in the combination group, their number was not significantly decreased (**Figure 7D**). On the other hand, in well-developed B16-F10 tumors, the number of CD8^+^ T cells was low and about 40% of them expressed PD-1 (**Figures 7E, F**). Tumors injected with cGAMP showed an over fourfold increase of CD8^+^ T cells in the TME, but combination with CA4P did not alter the number of tumor-infiltrating CD8^+^ T cells compared with the control tumors. However, the majority (over 70%) of tumor-infiltrating CD8^+^ T cells in the cGAMP group exhibit an exhausted CD8^+^PD-1^+^ phenotype. The number of CD69^+^ T cells was also elevated following STING agonist stimulation, indicating CD8^+^ T-cell exhaustion (**Figures 7G, H**).

These data suggest that in MHC I^+^ tumors with low levels of STING protein, cGAMP injection is not sufficient to induce effective CD8^+^ T-cell response. In contrast, cGAMP injection mobilizes CD8^+^ T cells to MHC I-deficient tumors with high levels of STING protein; however, they are masked by PD-1 expression.

### Combined therapy reduces resistance to anti-PD-1 therapy of poorly responsive tumors

Given that we have observed the TME shifting of non-immunogenic tumors into more immunogenic, we explored the cGAMP and CA4P combination to enhance the therapeutic efficacy of anti-PD-1 therapy of PD-1-resistant 4T1 and B16-F10 tumors. Therefore, mice with well-developed tumors were treated with cGAMP, CA4P, and monoclonal anti-PD-1 antibody, as shown in the diagram (**Figure 2A**). 4T1 tumors showed resistance to anti-PD-1 therapy (<30% of tumor growth inhibition, TGI). Triple combination failed to improve the benefit over the cGAMP and CA4P double combination (60% TGI compared with the control group 7B). B16-F10 melanoma was classified as resistant to anti-PD-1 therapy (11, 12), whereas in our study, a partial response on the 21st day of the therapy is shown (70% TGI without evidence of complete response) (**Figure 2E**). Indeed, we have observed a rapid tumor growth following the retardation phase. In B16-F10 tumors, triple combination improved the benefit over cGAMP monotherapy and cGAMP+CA4P double therapy (over 99% TGI compared with control in the triple combination versus 75% TGI compared with control in the double combination). Consistent with our previous results, combination with an anti-vascular agent did not bring additional significant benefit. Combination of the PD-1 checkpoint inhibitor with cGAMP and CA4P resulted in a complete tumor response in two B16-F10 tumor-bearing mice (CR: 2/5) and two partial responses (PR: 2/5). Combination of the PD-1 checkpoint inhibitor with cGAMP only resulted in one complete tumor response (CR: 1/5) and three partial responses (PR: 3/5).

These data suggest that the effectiveness of combining anti-PD-1 treatment with a STING agonist and/or an anti-vascular agent depends on the tumor microenvironment context. The STING agonist and anti-vascular agent in 4T1 tumors dampened the level of CD8^+^PD-1^+^ T cells; therefore, PD-1 blockade did not improve the therapeutic effect of combination therapy. In contrast, in B16-F10 tumors, the STING agonist induced massive CD8^+^PD-1^+^ T-cell influx; hence, additional benefit upon anti-PD-1 treatment could be observed.

## Discussion

Vascular disrupting agents (VDAs), including CA4P, exhibit limited single-agent antitumor activity (1, 13). Preclinical studies have shown that CA4P indeed disrupts existing tumor vasculature, especially in the core of the tumor, which is often resistant to conventional chemotherapy and radiation (1). However, its therapeutic benefit may be limited due to a remaining viable rim of tumor cells. Without additional treatment, the tumor can rapidly vascularize and regrow (14). Indeed, we have observed constant growth of tumors treated with CA4P as monotherapy, and the volume of these tumors was comparable with the control tumors at the end of treatment (**Figure 1**). Therefore, in clinical trials (15), CA4P has been combined with other treatments, such as antiangiogenic therapy (anti-VEGF antibody bevacizumab (16, 17)), chemotherapy (paclitaxel, carboplatin (18)), and radiotherapy (19). In those trials, CA4P has been well tolerated and has demonstrated therapeutic benefit over standard treatment regimens. Only few studies tested the combination of CA4P with immunotherapy. Recently, CA4P has been combined with CAR-T-cell therapy. Deng et al. have shown that CA4P in combination with CAR-T cells greatly increased the antitumor ability of CAR-T cells in preclinical models of solid human tumors (2). Shen et al. have shown that combination of CA4P with vascular endothelial growth receptor 2 inhibitor (DC101) improved anti-PD-1 therapy in a preclinical study of murine hepatocellular carcinoma (H22) (8). Another preclinical study investigated the ability of the CA4P analogue (Oxi4503) to improve the responsiveness to immune checkpoint inhibitors (ICIs, namely, PD-1, PD-L1, CTLA-4) of resistant murine mammary carcinoma (C3H) (3). Therefore, our study covered the need of examination of the antitumor effect of CA4P in combination with an immune stimulating agent.

STING protein has become a promising target of interest in the field of cancer immunotherapy. Activation of the cGAS-STING pathway induces the production of type I interferons and other proinflammatory cytokines and elicits and/or boosts potent antitumor immune response (20). Many natural and synthetic STING agonists have been developed and tested in both preclinical and clinical settings (20, 21), but preliminary clinical results demonstrated limited antitumor efficacy (22, 23).

To our knowledge, this is the first study to report application of cGAMP and CA4P as combined therapy and to show extremely different results of applied treatment, which strictly depend on the TME composition. We have observed that a synergistic effect of two agents in combination was observed only in 4T1 tumors. In B16-F10 tumors, STING activation was sufficient to achieve efficient tumor growth inhibition whereas CA4P did not confer any additional therapeutic benefit. High expression of STING protein by both tumor and endothelial cells may provoke efficient antitumor response. Demaria et al. have revealed the principal role of the tumor vasculature in the initiation of antitumor response via STING signaling. They showed that in response to cGAMP injection, endothelial cells were a principal source of type I IFN (7). However, our data indicate that immune response following STING activation was not strong enough to elicit potent and sustained antitumor response in 4T1 tumors. It has been reported that in a wide variety of cancers, STING signaling can be suppressed by epigenetic silencing of cGAS or STING itself (24). Therefore, combination with an agent that destroys existing tumor blood vessels was a rationale in the case of highly vascularized 4T1 tumors. Combination with CA4P, in addition to its vascular destructive benefits, could also strengthen the antitumor response via cGAS-STING activation.

Intratumoral injection of the STING agonist activates innate immune and stimulates reversion of immunosuppression to boost anticancer response in non-immunogenic tumors. Despite proven therapeutic efficacy in some preclinical models, repolarization of the suppressive tumor milieu is still poorly studied (25). We have shown that in both tumor models, repolarization of macrophages from immunosuppressive M2 toward the M1 immunostimulatory phenotype was efficient after combination therapy. Depending on the type of macrophages within the tumor mass, TME status may be determined as “cold” (which consists of TAMs M2) or “hot” (with TAMs M1) (26). Polarization of macrophages from M2 to M1 phenotype plays an important role in the antitumor effects of the STING signaling (27). Studies of others also support the notification that the STING agonist can switch a “cold” TME into a “hot” one via tumor-infiltrating macrophage repolarization (28, 29). Ohkuri et al. have shown that CD11b^+^Ly6C^high^ proinflammatory macrophages are recruited to the tumor in a STING-dependent manner (30, 31). Ager et al. presented a role of STING agonist in repolarization of suppressive myeloid populations in mice and humans, conferring effective immunotherapy (25). Taken together, our and other results indicate that the STING-triggered tumor-accumulating and repolarized macrophages participate in the antitumor effects of the STING-activating compound.

Recent data demonstrate powerful antitumor response in numerous cancer models, mediated by NK cells induced by therapeutic application of STING agonists (32–35). Stimulation of the STING pathway can overcome barriers to immunotherapy response such as immune exclusion and exhaustion and is a very promising immune stimulation strategy to eradicate cancers (36). According to our and other data, STING agonist administration effectively stimulates NK cells to destroy tumors. Depletion of NK cells abolishes the therapeutic effect of the STING agonist, which indicates their key role in anticancer therapy (37). We observed significantly enhanced recruitment of NK cells to the TME following combination therapy in both tumor models (**Figure 6**). It has been reported that TAMs have a role in altering NK-cell function and phenotype. TAM M2 macrophages substantially inhibited NK-cell activation and cytotoxicity against tumor cells (38). In contrast, TME modulation via IL-12 and anti-TGFβ increase the maturity and the level of activation markers of tumor-associated NK cells (39).

The mechanism of killing cancer cells by NK cells is MHC I dependent. NK cells recognize and attack cells lacking MHC I molecules. Activated NK cells infiltrating B16-F10 tumors could efficiently eliminate MHC I-deficient cancer cells. In 4T1 MHC I-positive tumors, NK cells could have also eliminated cancer cells but probably by a distinct mechanism of action. Cellular stress associated with applied treatment (e.g., DNA damage response upon CA4P treatment) could upregulate stress-associated molecules (“induced-self” ligands) which act as ligands for NK activating receptors. Upregulated ligands permit NK-cell activation by overcoming MHC class I-dependent inhibition. As a result, tumor cells can be directly eliminated through NK cell-mediated cytotoxicity or indirectly through secretion of proinflammatory cytokines (40).

Initially, the antitumor effect of STING therapy has been attributed to CD8^+^ T-cell response (7, 41, 42). However, in the 4T1 model, we did not notice an elevated level of tumor-infiltrating CD8^+^ T cells (TILs) following applied treatments. In melanoma instead, only cGAMP injection induced massive CD8^+^ T-cell infiltration, turning the melanoma milieu into “hot” infiltrated-inflamed tumors, characterized by high TIL level-expressing immune checkpoint receptor PD-1 (**Figure 7**) (26). We also found that CD8^+^ T cells infiltrating tumor tissue express CD69 to some extent and its expression correlate with PD-1. CD69 is known to be a marker of early leukocyte activation. It is also known to be expressed on resident memory T cells, playing a crucial role in recruitment and retention of T cells in inflamed tissue. Several papers indicated the important role of CD69 in inducing the exhaustion of tumor-infiltrating T cells. Mita et al. have shown that tumor growth and metastasis were attenuated in *Cd69^-/-^* mice. That inhibition was associated with increased T-cell infiltration and reduced CD8^+^ T-cell exhaustion (10). Others indicated that CD69 expression is a characteristic feature of resident, terminally exhausted CD8^+^ T cells (43, 44).

Both tested tumor models were classified as poorly immunogenic, “cold” tumors, weakly responding to immune checkpoint inhibitors (ICI-resistant tumors) (11, 12). STING agonist therapy is recognized to increase influx of tumor-infiltrating CD8^+^ T cells and therefore converting immunologically “cold” tumors into “hot” tumors, increasing responsiveness to ICI treatment. CD8^+^ T cells are crucial for effectiveness of PD-1 blockade; however, MHC I molecules on tumor cells seem to be a necessary condition for a successful ICI treatment (45). MHC I is a ligand for TRC, and its recognition leads to T-cell activation. In B16-F10, MHC I-deficient melanoma, cGAMP triggered massive influx of CD8^+^ T cells to the tumor. When testing PD-1 blockage, we have observed an efficient ICI treatment response with cases of complete tumor remission (**Figure 2**). The reason for this phenomenon may be MHC I upregulation by B16-F10 tumor cells under the influence of INFγ produced by cGAMP-activated immune cells, including NK cells (46, 47). 4T1 MHC I-positive tumors exhibited PD-1 blockade resistance. CD8^+^ T-cell influx triggered upon STING activation was too low, and they themselves did not exhibit elevated levels of PD-1. It has been shown in cGAS- or STING-deficient mice that anti-PD-1 treatment failed to induce antitumor effects. The authors indicated that cGAS-STING signaling may need to be screened in patients before STING agonist application in combination with ICI (48). The efficacy of ICI was substantially increased in tumor models when combined with cGAMP in a cGAS-STING signal-sufficient context (49).

## Conclusions

Our results indicate that the TME needs to be carefully screened before applying antitumor treatment. The TME greatly influences the outcome of the therapy, including immune cell contribution and ICI responsiveness. The evaluation of the STING protein level is necessary to conduct and expect satisfactory results with the treatment of STING agonists. MHC I expression should also be a key feature checked by the therapy application. Additional treatments could be considered to upregulate MHC I molecules in order to achieve successful response to immune checkpoint blockade.

## Data availability statement

The original contributions presented in the study are included in the article/**Supplementary Material**. Further inquiries can be directed to the corresponding authors.

## Ethics statement

Experiments on animals were carried out in accordance with standard procedures, with the consent of the Local Ethics Commission of Animal Experiments in Katowice (permission no. 22/2021). Animals were treated in accordance with the recommendations in the Guide for the Care and Use of Laboratory Animals of the National Institutes of Health. Experiments on animals were conducted in accordance with the 3R rule.

## Author contributions

RS and JC conceptualized the experiments. JC, AD, SM, TC, MR, EP, MJ-B, and RS performed the experiments. JC, RS, AD, and SM analyzed the data. JC wrote the manuscript. RS and AD revised the manuscript. All authors contributed to the article and approved the submitted version.
