# Supplementary material for: Antitumor effect of anti-vascular therapy with STING agonist depends on the tumor microenvironment context
Source: Front Oncol. 2023 Aug 15;13:1249524. doi: 10.3389/fonc.2023.1249524 (PMC10465696; doi:10.3389/fonc.2023.1249524)
Supplement: Supplementary file 1 [file DataSheet_1.docx]

Materials and Methods

1. Cell lines:

Murine melanoma B16-F10 cell line and 4T1 breast cancer (ATCC, Manassas, WV, USA) were cultured in RPMI 1640 (Thermo Fisher Scientific, Waltham, MA, USA) supplemented with 10% heat-inactivated fetal bovine serum (Thermo Fisher Scientific,Waltham, MA, USA). Cell cultures were incubated in standard conditions (37°C, 5% CO2, 95% humidity). Cells were passaged every 3–4 days.

1. Western blot analysis:

The cells were harvested by trypsinization, washed with PBS and the cell pellets were frozen at -80^o^C. The cells were lysed by addition (to the frozen pellet) of the IP buffer (50 mM Tris-HCl, pH 8.0; 120 mM NaCl; 0.5% NP40) supplemented with the protease inhibitors (PMSF, pepstatin A, aprotinin, and leupeptin) and phosphatase inhibitor cocktail 2 (Merck, Sigma, Darmstadt, Germany). Aliquots of lysates (50µg) were separated by SDS-PAGE on 8% gel and electro-transferred onto PVDF membrane. Before incubation with primary antibody, the membranes were incubated for 1 h at room temperature in blocking solution (5% skim milk in PBS with 0.1% Tween-20). The incubations with the primary antibodies diluted in blocking solution were performed at 4^o^C overnight with gentle rocking. The following primary antibodies were used: anti-STING (clone: D2P2F) from Cell Signaling Technology (Danvers, MA, USA), anti-HSC70 (clone: B-6) from Santa Cruz Biotechnology (Dallas, TX, USA). HRP-conjugated secondary antibodies (anti-mouse or anti-rabbit) were detected by chemiluminescence (SuperSignal West Pico substrate; Thermo Fisher Scientific, Waltham, MA, USA).

1. Therapeutic agents:

Cyclic guanosine monophosphate–adenosine monophosphate (cyclic GMP-AMP, 2′3′-cGAMP, #vac-nacga23, Invivogen, Toulouse, France) was injected intratumorally in a dose 5 µg/mouse (in 100µl PBS¯). Combretastatin A4 Phosphate Disodium (CA4P, Fosbretabulin, #S7204, Selleckchem, Planegg, Germany) was injected intraperitoneally at a dose of 50 mg/kg body weight in 100µl saline/mouse. Monoclonal anti-PD-1 antibody (#114119, clone: RMP1-14, Biolegend, San Diego, CA, USA) in a dose 200µg in 100µl PBS¯/mouse was injected intraperitoneally.

1. Therapy:

8 to 10-week-old, female C57Bl/6NCrl (n=85) or BALB/c (n=105) mice were injected subcutaneously into lower flank with 2x10^5^ B16-F10 cells or 2x10^5^ 4T1 cells in 100µL PBS¯, respectively. Tumors were measured with calipers one or two days. Tumor volumes were determined using the formula: volume = width^2^ x length x 0.52. When tumors reached approximately 50mm^2^ (7 days after inoculation for 4T1 tumors, 10 days for B16-F10) mice were randomly divided into four treatment groups: Control, cGAMP, CA4P, cGAMP + CA4P. All treatment groups include n=5 mice. Experiments were not blinded. Each mouse was labeled and placed in signed cages to avoid confounders. Mice were allocated in order to obtain similar average tumor volume in the treatment groups. All mice intended to the experiments were used. The inclusion criterion was a developed tumor. There were no exclusions. Therapy was repeated 4 times on BALB/c mice and 3 times on C57Bl/6NCrl mice. Animals were treated according to group allocation: the control group left untreated, the cGAMP group with two doses of cGAMP in a four-day interval, the CA4P group two doses of CA4P in a four-day interval, the cGAMP + CA4P group with two doses of cGAMP and CA4P in a four-day interval with a one-day shift. The schedule of therapeutic agents administration is shown in Figure 2. In an experiment combining immune checkpoint inhibitor anti-PD-1, mice were divided randomly into five treatment groups, 5 mice in each group: Control, PD-1, cGAMP+PD-1, CA4P+PD-1, cGAMP+CA4P+PD-1. Mice received cGAMP and CA4P as described above, anti-PD-1 antibody was administered 3 times (in BALB/c mice) and 4 times (in C57Bl/6NCrl mice) in three- to four-day interval. The schedule is shown in Figure 1.

1. Immunofluorescence staining:

On the 14^th^ and 17^th^ day after 4T1 and B16-F10 cells inoculation, respectively, mice from all groups (n=5 in each group) were sacrificed by cervical dislocation and tumors were collected for further analyses. The collected tumors were embedded in OCT (Leica Biosystems, Wetzlar, Germany), frozen in liquid nitrogen and stored at −80 ^◦^C until needed. The tumors were further sectioned into 5 µm slices. Blood vessels were stained with anti-CD31 antibody (Abcam, Cambridge, UK) and subsequently with Alexa Fluor 594 conjugated secondary antibody (Abcam). Macrophages in the tumor sections were stained with anti-F4/80 (Abcam) followed by Alexa Fluor 488-conjugated secondary antibody (Abcam) and Alexa Fluor 594 CD206 antibody (BioLegend). Activated NK cells were identified using anti-Nkp46 antibody (BioLegend) followed by Alexa Fluor 594 conjugated secondary antibody. Microscopic observations were performed using a LSM710 confocal microscope (Carl Zeiss Microscopy GmbH). The obtained confocal images were analyzed with ImageJ 1.48v (National Institutes of Health, Bethesda, MD, USA) and the results were expressed as the percentage of area [%].

1. Immunohistochemical staining:

4T1 and B16-F10 tumors frozen sections were fixed with 4% PFA and quenched with 0,3% H_2_O_2_. HIER was performed using antigen unmasking solution (Vector Laboratories, H-3300, Burlingame, CA, USA). Tumor slices were blocked with ultravision protein block (Epredia, Breda, Netherlands) and then incubated with primary anti-STING antibody (Cell Signaling Technology, Danvers, Massachusetts, USA). DAB (Vector Laboratories, Burlingame, CA, USA) was used to visualize antigen. The analysis of the specimens was conducted using the Nikon Eclipse 80i microscope (Nikon Instruments Inc., Tokyo, Japan).

1. Flow cytometry analysis:

On the 14^th^ and 17^th^ day after 4T1 and B16-F10 cells inoculation, respectively, mice from all groups were sacrificed by cervical dislocation and tumors were collected for flow cytometric analyses. To obtain single-cells suspension collected tumors were digested with collagenase II solution (500U/ml; Gibco BRL, Paisley, UK). Red blood cells were lysed using ACK solution (Lonza, Basel, Switzerland). The obtained cell suspension was filtered using 70-μm and 40-μm cell strainers. Single cells suspension of mononuclear cells were selected by centrifugation with Histopaque-1077 gradient (Merck, Darmstadt, Germany). The isolated cells were blocked with anti-mouse CD16/32 antibody (BioLegend) and then incubated for 30min at 4^◦^C with antibodies directed against the following mouse antigens. The subpopulations of T lymphocytes were identified using: anti-CD45, anti-CD8, anti-CD69, anti-PD-1 antibodies (BioLegend). The level of NK cells was determined using: anti-CD45, anti-CD49b, anti-NKp46, anti-CD69 antibodies (BioLegend). Macrophages were identified using: anti-CD11b, anti-F4/80, anti-CD86, anti-CD206 antibodies (BioLegend). Dead cells were eliminated by using the viability dye 7AAD (Biolegend). In flow cytometric analyses (BD FACSCanto; BD, Franklin Lakes, NJ, USA), gates dividing negative from positive cells were based on isotype antibody control probes. Flow cytometry data were presented as the number of total immune cells subpopulation obtained from mononuclear cells and divided by tumor weight [mg].

1. Statistics

The normality of distribution was verified by the Shapiro-Wilk test. The homogeneity of variance was verified by the Levene test or the Brown-Forsythe test. For variables meeting the conditions of parametric tests, analysis of variance (ANOVA) with post-hoc Tukey HSD or LSD tests was performed. Those variables are shown as mean ± SEM; statistically significant differences are marked with an asterisk (*p<0.05; **p<0.01; ***p<0.001). For variables not meeting the conditions of parametric tests, the Kruskal-Wallis test was performed and variables are presented as median ± interquartile; statistically significant differences are marked with a box (#p<0.05; ##p<0.01; ###p<0.001). All statistical comparisons were performed using Statistica 13 software (StatSoft Inc.) and Prism V.9.0 Software (GraphPad Software, La Jolla, California, USA).

Table1. List of antibodies used in flow cytometry. To each antibody appropriate isotype antibody control was used.

| Antibody | Clone | Source | Identifier |
| --- | --- | --- | --- |
| CD16/32 | 93 | BioLegend | Cat# 101302 |
| CD45 FITC | 30-F11 | BioLegend | Cat# 103108 |
| CD49b PE | DX5 | BioLegend | Cat# 108908 |
| CD8 APC | 53-6.7 | BioLegend | Cat# 100712 |
| PD-1 PE/Cyanine7 | 29F.1A12 | BioLegend | Cat# 135216 |
| CD69 PE/Cyanine7 | H1.2F3 | BioLegend | Cat# 104512 |
| Nkp46 APC | 29A1.4 | BioLegend | Cat# 137608 |
| CD11b FITC | M1/70 | BioLegend | Cat# 101206 |
| F4/80 PE/Cyanine7 | BM8 | BioLegend | Cat# 123114 |
| CD206 PE | C068C2 | BioLegend | Cat# 141706 |
| CD86 APC | GL-1 | BioLegend | Cat# 105012 |

Table1. List of antibodies used in immunofluorescence staining.

| Antibody | Clone | Source | Identifier |
| --- | --- | --- | --- |
| CD31 | MEC 7.46 | Abcam | Cat# ab7388 |
| CD206  (Alexa Fluor® 594) | C068C2 | BioLegend | Cat# 141726 |
| CD335 (Nkp46) | 29A1.4 | BioLegend | Cat# 137601 |
| F4/80 | CI:A3-1 | Abcam | Cat# ab6640 |
| Goat Anti-Rat IgG H&L (Alexa Fluor® 488) | Polyclonal | Abcam | Cat# ab150157 |
| List of antibodies used in immunohistochemical staining | | | |
| STING | D2P2F | Cell Signaling Technology | Cat# 13647 |
| Goat anti-mouse/rabbit IgG HRP detection system | Polyclonal | ImmunoLogic | Cat# DPVB110HRP |

Supplementary figures

| 4T1 | B16-F10 |
| --- | --- |
| * | **  ** |

Suppl. Fig. 1 Total CD8+ T cells infiltration in 4T1 and B16-F10 tumors. The number of all CD8+ T cells obtained from total single cell-suspensions were calculated per 1 mg of tumor tissue. Results from three replicate experiments are shown, n=5-15 for each group. Data are shown as mean ± SEM for *p<0.05 by Anova with post-hock LSD Tests or **p<0.01 by Kruskal–Wallis multiple comparisons.

**A)**


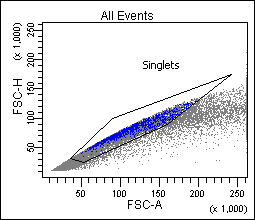

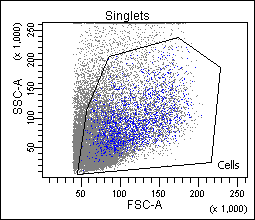

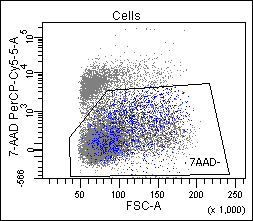

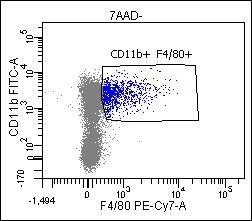

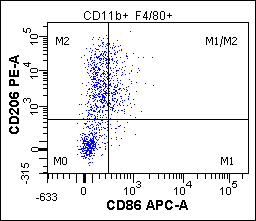


**B)**


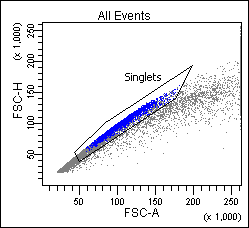

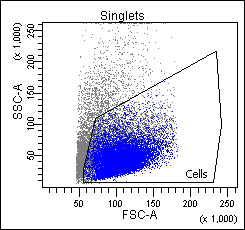

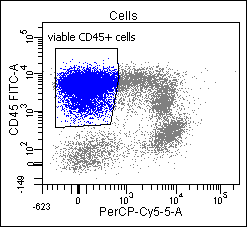

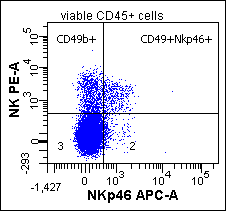

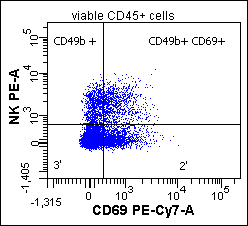

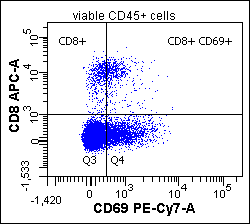

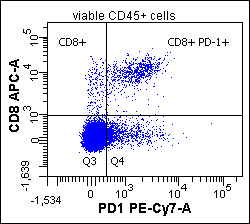


5a.

5b.

I.

II.

III.


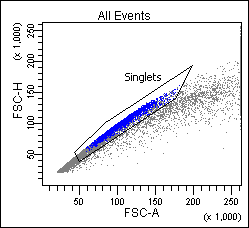

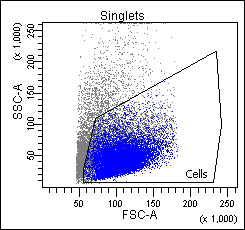

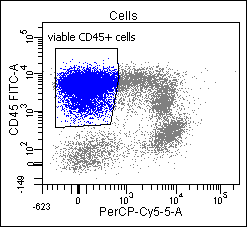

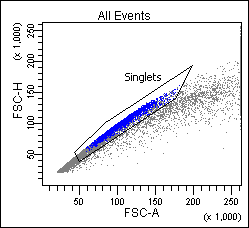

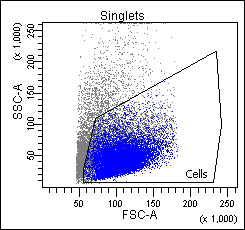

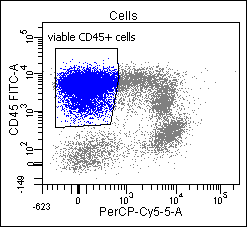


Suppl. Fig. 2 Gating strategy for flow cytometry analysis. Flow cytometry was performed on two laser BD FACSCanto cytometer. **A)** Gating strategy for macrophages populations, M1: 7AAD-CD11b+F4/80+CD206-CD86+ and M2: 7AAD-CD11b+F4/80+CD206+CD86-. **B)** **I:** Gating strategy for activated NK cells: 7AAD-CD45+CD49b+Nkp46+; **II:** for CD8 T cells and NK cells: 7AAD-CD45+CD8+CD49b+CD69+; **III:** for CD8 T cells: 7AAD-CD45+CD8+PD-1+. All gates were set to isotype IgG control for each sample separately.
